# Nucleus Accumbens Shell Orexin-1 Receptors Are Critical Mediators of Binge Intake in Excessive-Drinking Individuals

**DOI:** 10.3389/fnins.2019.00088

**Published:** 2019-02-13

**Authors:** Kelly Lei, Claudina Kwok, David Darevsky, Scott A. Wegner, JiHwan Yu, Lisa Nakayama, Vincent Pedrozo, Lexy Anderson, Shahbaj Ghotra, Mary Fouad, Frederic W. Hopf

**Affiliations:** Alcohol and Addiction Research Group, Department of Neurology, University of California, San Francisco, San Francisco, CA, United States

**Keywords:** alcohol, nucleus accumbens, shell, orexin, SB-334867, excessive drinkers, binge

## Abstract

Excessive, binge alcohol drinking is a potent and pernicious obstacle to treating alcohol use disorder (AUD), and heavy-drinking humans are responsible for much of the substantial costs and harms of AUD. Thus, identifying key mechanisms that drive intake in higher-drinking individuals may provide important, translationally useful therapeutic interventions. Orexin-1-receptors (Ox1Rs) promote states of high motivation, and studies with systemic Ox1R inhibition suggest a particular role in individuals with higher intake levels. However, little has been known about circuits where Ox1Rs promote pathological intake, especially excessive alcohol consumption. We previously discovered that binge alcohol drinking requires Ox1Rs in medial nucleus accumbens shell (Shell), using two-bottle-choice Drinking-in-the-Dark (2bc-DID) in adult, male C57BL/6 mice. Here, we show that Shell Ox1Rs promoted intake during intermittent-access alcohol drinking as well as 2bc-DID, and that Shell inhibition with muscimol/baclofen also suppressed 2bc-DID intake. Importantly, with this large data set, we were able to demonstrate that Shell Ox1Rs and overall activity were particularly important for driving alcohol consumption in higher-drinking individuals, with little overall impact in moderate drinkers. Shell inhibition results were compared with control data combined from drug treatments that did not reduce intake, including NMDAR or PKC inhibition in Shell, Ox1R inhibition in accumbens core, and systemic inhibition of dopamine-1 receptors; these were used to understand whether more specific Shell Ox1R contributions in higher drinkers might simply result from intrinsic variability in mouse drinking. Ineffectiveness of Shell inhibition in moderate-drinkers was not due to a floor effect, since systemic baclofen reduced alcohol drinking regardless of basal intake levels, without altering concurrent water intake or saccharin consumption. Finally, alcohol intake in the first exposure predicted consumption levels weeks later, suggesting that intake level may be a stable trait in each individual. Together, our studies indicate that Shell Ox1Rs are critical mediators of binge alcohol intake in higher-drinking individuals, with little net contribution to alcohol drinking in more moderate bingers, and that targeting Ox1Rs may substantially reduce AUD-related harms.

## Introduction

Excessive, binge-level alcohol consumption is a major obstacle when treating alcohol use disorder (AUD) (Harwood et al., 1998; Larimer et al., 1999; Blincoe et al., 2002; Mokdad et al., 2004; Dawson et al., 2005; Hingson et al., 2005; Rehm et al., 2009; Koob and Volkow, 2010; Bouchery et al., 2011; Sacks et al., 2013; Center for Disease Control and Prevention [CDC], 2014; Substance Abuse and Mental Health Services Administration [SAMHSA], 2014). Importantly, heavy-drinking individuals consume a substantial proportion of the significant costs and harms of AUD (e.g., Center for Disease Control and Prevention [CDC], 2014). Thus, there is considerable importance in identifying mechanisms that promote excessive binging in these higher drinkers, especially considering the limited availability of pharmacotherapies whose efficaciousness is restricted to a subset of AUD patients (Spanagel, 2009; World Health Organization [WHO], 2014).

Orexin signaling has been identified as being of particular importance for driving many motivation- and addiction-related behaviors (Mahler et al., 2012, 2014; Boutrel et al., 2013; Barson and Leibowitz, 2016; James et al., 2017). Orexin is synthesized in lateral hypothalamus cells which project broadly across the brain (de Lecea et al., 1998) and contribute to a wide variety of regulatory and homeostatic behaviors (Mahler et al., 2014; Brown et al., 2015; Li et al., 2016; James et al., 2017). Orexin-1-type receptors (Ox1Rs) in particular contribute to highly motivated behaviors, including intake of natural rewards and intoxicants such as alcohol (Borgland et al., 2009; Moorman and Aston-Jones, 2009; Cason et al., 2010; Anderson et al., 2014; Baimel et al., 2014; Barson et al., 2014; Mahler et al., 2014). Thus, Ox1Rs could be a novel intervention to treat AUD and other motivational disorders (Khoo and Brown, 2014; Li et al., 2016).

Importantly, some studies using systemic Ox1R inhibition have suggested that Ox1Rs are particularly important for promoting alcohol intake in higher-drinking individuals (Moorman and Aston-Jones, 2009; Alcaraz-Iborra et al., 2017; Moorman et al., 2017). However, the brain circuit in which Ox1Rs act to drive consumption in higher drinkers remains unknown. We recently demonstrated that binge alcohol intake requires Ox1Rs in the medial nucleus accumbens (NAc) Shell (Shell) (Lei et al., 2016b), a brain region that helps regulate a variety of motivation- and addiction-related behaviors (Anderson et al., 2008; Chaudhri et al., 2010; Saddoris et al., 2013; Castro et al., 2015; Corbit and Balleine, 2015; Marchant et al., 2015; Millan et al., 2015) including alcohol consumption (Kasten and Boehm, 2014; Lum et al., 2014; Wilden et al., 2014; Ramaker et al., 2015).

To help develop a larger data set to robustly understand whether Shell Ox1Rs underlie excessive consumption in higher-drinking individuals, we examined intake of 15% alcohol under a two-bottle-choice variant of Drinking-in-the-Dark paradigm (2bc-DID) that we have previously utilized (Lei et al., 2016a,b). We also examined the importance of Shell Ox1Rs across individual in another model which also leads to excessive intake of alcohol, 20% alcohol intake under an two-bottle-choice intermittent access model (2bc-IA) (Hwa et al., 2011; Morisot et al., 2018) (see section “Materials and Methods”). We demonstrate that either Ox1R blockade or GABAR-mediated inhibition of activity in the Shell significantly reduces alcohol consumption in excessively-drinking subjects, but with no overall effect in more moderate-drinking mice. To better understand the relationship between Shell inhibition and effects on drinking across individuals, we also developed a control group consisting of several pharmacological agents which did not alter drinking, including studies in the vmPFC since our previous work found that vmPFC Ox1Rs also promote 2bc-DID drinking (Lei et al., 2016b). Together, we show that Shell Ox1Rs are critical promoters of increased intake in higher-drinking individuals, and might represent a potent translational target to reduce the harms of human binge alcohol intake and AUD.

## Materials and Methods

### Animals

All procedures followed the Guide for Care and Use of Laboratory Animals provided by the National Institutes of Health, and with approval of the Institutional Animal Care and Use Committee of UCSF. Male C57BL/6 mice, 6–8 week of age, were purchased from Jackson Laboratories. Animals were single-housed under a reverse 12:12 light:dark cycle, with lights off at 10:00 a.m. Food and water were available, *ad libitum*, for all subjects.

### Two-Bottle Choice Drinking-in-the-Dark (2bc-DID) for Alcohol or Saccharin

We used a modified drinking in the dark paradigm as previously described (Lei et al., 2016a,b,c). After ∼2 weeks acclimation, mice were first given 24 h two-bottle choice access to 15% alcohol (v/v) and water. Thereafter, mice drank under a limited daily access paradigm, where they were given two-bottle choice of 15% alcohol and water for 2 h/day. 5 day/week starting at ∼3 h after lights off. To test for specificity of drugs on alcohol drinking, a subset of mice drank a 0.05% saccharin solution an identical schedule to that used for alcohol; this concentration was previously determined to yield a similar volume of intake as alcohol (Lei et al., 2016a,b).

### Two-Bottle Choice Intermittent Access (2bc-IA) for Alcohol

After ∼2 weeks acclimation, mice drank under an intermittent access schedule where they received overnight (∼24 h) two-bottle choice access to 20% alcohol (v/v) and water starting on Monday, Wednesday, and Friday at ∼3 h after lights off (Hwa et al., 2011; Morisot et al., 2018).

### Cannula Implantation Surgeries

After ∼2-week of alcohol access under 2bdc-DID or 2bc-IA, surgery was performed to bilaterally implant guide cannulae (Plastics One) aimed at either the Shell (AP +1.5, ML ± 0.5, and DV -4.8 mm), NAc Core (AP +1.5, ML ± 1.0, and DV -4.0 mm) or vmPFC (AP +1.7, ML ± 0.4, and DV -2.7 mm). All given coordinates are relative to Bregma from skull surface. After surgery, mice were allowed to recover for at least 3 days before alcohol drinking was resumed. At the end of experiments, brains were harvested for histology and cannula placement verification.

### Drug Treatment

Agents, doses, and relevant references for the doses used are given in Table 1. The selective OX1R antagonist, SB-334867 (Tocris) and the PKC inhibitor, chelerythrine (Abcam), were dissolved in 100% DMSO. OrexinA peptide (Sigma), the selective OX2R antagonist, TCS-OX2-29 (TCS, Tocris), and the NMDAR antagonist, AP5 (Tocris) were dissolved in 0.9% saline, as was the D1R selective antagonist, SCH-23390 (SCH, Sigma). A cocktail of the GABAA agonist muscimol (Sigma) and GABAB agonist baclofen (RBI) (M/B) were dissolved in DMSO, and 50 ng/side each was injected intracranially. Some mice were injected with 1 or 5 mg/kg baclofen i.p., dissolved in saline. The compounds we tested have been widely used and thus we tested a single dose taken from literature. Also, although the SCH23390 dose is high, it has been previously shown to be selective in that it reduces acquisition of alcohol CPP without altering LiCl CPA (Pina and Cunningham, 2014). While 100% DMSO is a high dose for intracranial, we (Lei et al., 2016b) and other groups have used this intracranial vehicle (Pierce et al., 1999; James et al., 2011; Simms et al., 2011; Vendruscolo et al., 2015). Importantly, our studies were performed with a randomized, Latin-squares design, with alcohol drinking on days in between intracranial test sessions, and any possible lingering toxicity of DMSO should impact drinking on subsequent days, which was not observed. We also note that other studies have used concentrations of SB that are much lower from what we utilize, e.g., where Thorpe and Kotz (2005) used 6 ng in Shell to block orexin enhancement of feeding. However, this paper uses aCSF as the vehicle (see also Zheng et al., 2007). In recent times, many studies, including our own, use a DMSO-based strategy (e.g., see James et al., 2011), and we and others do not get SB solubility in aCSF. We also note that comparison of doses with older studies may be challenging given observations that there can be batch-related differences in SB solubility and color (Jeff Simms, personal communication, a co-author from Richards et al., 2008, and other orexin-related publications).

**Table 1:** Pharmacological agents, doses used, and applicable references.

Agent	Dose	Reference
SB-334867	3-μg/side, i.c.	Hollander et al., 2008; Espana et al., 2010; Plaza-Zabala et al., 2012; Lei et al., 2016b
Muscimol/baclofen	50 ng/side for each, i.c.	Chaudhri et al., 2010; Millan et al., 2010; Wilden et al., 2014
Baclofen	1, 5 mg/kg, i.p.	Crabbe et al., 2017
Chelerythrine	0.03–0.4 μg/side, i.c.	Cervo et al., 1997; Narita et al., 2004
SCH23390	0.3 mg/kg, i.p.	Pina and Cunningham, 2014
OrexinA	100 pmol/side, i.c.	Thorpe and Kotz, 2005; Mayannavar et al., 2014; Zajo et al., 2016
TCS-OX2-29	3-μg/side, i.c.	Brown et al., 2013; Qi et al., 2013; Lei et al., 2016b
AP5	0.3 μg/side. i.c.	David et al., 2004; Bergado Acosta et al., 2017


*i.c., indicates intracranial.*

All animals were habituated to handling and injection prior to drug treatment sessions. After 2–3 days of simple handling, animals have 2–3 days of handling where cannula plug is removed and returned; finally, animals have one saline injection prior to drug test sessions. Drug deliveries occurred 30 min prior to drinking. Drugs and their vehicles were injected in a counter-balanced manner in different cohorts of mice. For many cohorts, vehicle and drug were each tested twice in each animal in a counter-balanced, randomized fashion, and averaged to give a single vehicle intake value and single drug intake value for each animal. SCH, orexinA ± TCS, vmPFC AP5, and some SB and M/B in 2bc-DID were tested with only a single injection of vehicle and drug. There was at least one drinking day between drug treatments and not more than two injections per week. For i.p. delivery, drugs were injected at a volume of 10 mL/kg. For intracranial injections, drugs were bilaterally injected at a volume of 200 nL at a rate of 200 nL/min, with the exception of OXA, which was injected at a volume of 300 nL. The infusion needles (Plastics One) were left in place for an extra 60 s before retraction. For Shell and Core, needles projected 0.3 mm past end of guide cannulae, and for vmPFC needles projected 0.5 mm.

### Data Analyses

After each drinking session, alcohol (g/kg of body weight) or saccharin intake (mL/kg of body weight), water intake, and the preference ratio (volume of alcohol intake/total volume of fluid intake) were measured. Data were statistically analyzed using Prism (GraphPad), SPSS (IBM), and R v3.4.4 (R Foundation for Statistical Computing).

Alcohol drinking was analyzed with paired *t*-tests comparing vehicle vs. drug treatment within each animal. Non-normal data, including concurrent water intake, were tested with Wilcoxon match-paired signed rank test (for paired data) or Mann–Whitney test (for unpaired data). To compare the basal intake vs. change in drinking slope relationships across different groups, a one-way ANCOVA was performed with a dependent variable of Change in Drinking, an independent variable of Treatment Group, and a covariate of Basal Drinking. The presence of a statistically significant interaction between Treatment Group and Basal Drinking indicates a dissociable effect of Treatment Group on the slope of a regression line which examines Change in Drinking as a function of Basal Drinking. All bar data are shown as mean ± SEM.

We were particularly interested in understanding the relationship between basal drinking levels and the requirement for Shell Ox1Rs/activity for driving alcohol intake. Thus, we took advantage of the large data sets we had acquired to study a Shell inhibition group, which consisted of data combined from Ox1R or global GABA-mediated inhibition within the Shell. Since there was variability in mouse intake, we also compared Shell inhibition results with data combined from a series of control groups where no change was observed with drug treatment. This gave us a robust comparison to help account for intrinsic variability in mice drinking and allowed us to study whether a greater Shell contribution in higher-drinking individuals represented mathematical effects such as regression to the mean. Basal alcohol intake was normally distributed in both groups, and there was no difference in basal intake level between the Shell inhibition group and control group (*t*_202_ = 0.8891, *p* = 0.3750).

We first compared basal drinking levels with the actual change in alcohol intake with drug treatment. In addition, because lower basal drinkers have a smaller maximal change in drinking levels, we next normalized the drug-related drinking change to account for different basal intake levels in each animal. In particular, we calculated the log[100^∗^(intake during drug treatment)/(intake during vehicle)]. This allows us to express the impact of Shell inhibition or other drug treatment on drinking relative to baseline consumption levels, but also partially correct for the high percent increases that can be observed when lower basal intake levels go up. Using this measure, a log value of 2 (log[100]) indicates no intake change with treatment. For subjects where treatment reduced drinking to zero, the log was set to 1. This method thus reflected a useful compromise to examine the percent drop in drinking so that it could be compared with basal drinking levels across individuals. In addition, in order to examine the distribution of treatment effects across individuals (shown in Figure 2G,H), we determined the number of animals in each of the following bins of log[100^∗^(intake during drug treatment)/(intake during vehicle)] values: 1–1.3, >1.3–1.5, >1.5–1.7, >1.7–1.9, >1.9–2.1, >2.1–2.3, >2.3–2.5, >2.5.

## Results

### Shell Inhibition by Ox1R Blockers or GABA Agonists Decreases Alcohol Intake

Previous studies using systemic Ox1R inhibition have suggested that Ox1Rs promote intake in higher-drinking individuals (Moorman and Aston-Jones, 2009; Alcaraz-Iborra et al., 2017; Moorman et al., 2017). We previously demonstrated that Shell Ox1Rs promote 2bc-DID drinking (Lei et al., 2016b) (see section “Materials and Methods”), and that animals drinking under this model reach binge-level blood alcohol concentrations (Lei et al., 2016a). To help understand whether Shell Ox1Rs underlie excessive consumption in higher-drinking individuals, we examined intake under two different high alcohol drinking models, 2bc-DID and 2bc-IA (see section “Materials and Methods”). Drug versus vehicle were tested within each animal using a randomized, counterbalanced design.

**FIGURE 1:**
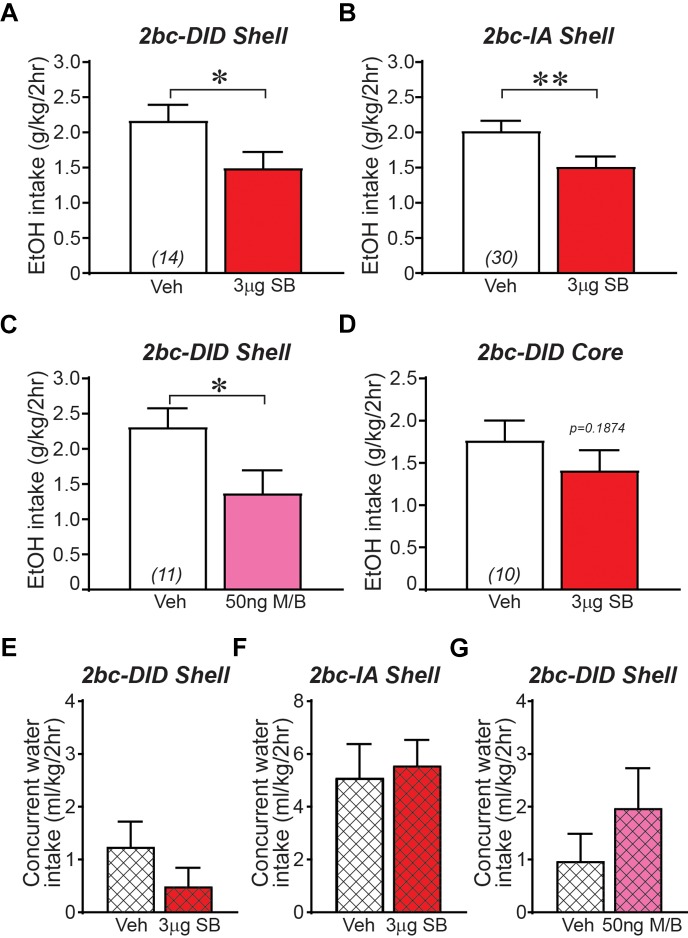
Ox1R blockade or GABAergic inhibition within medial NAc Shell significantly reduced alcohol drinking in mice. **(A)** Intra-Shell infusion of the Ox1R inhibitor SB reduced alcohol intake in the 2bc-DID model, replicating previous studies (Lei et al., 2016b). For this and all other bar graphs, open bar is with vehicle injection, shaded bar is with drug injection, tested within-animal. **(B)** Intra-Shell SB reduced alcohol intake in the 2bc-IA. **(C)** Global inhibition of Shell with GABA receptor agonists muscimol/baclofen significantly reduced 2bc-DID alcohol intake. **(D)** Ox1R inhibition in Core did not significantly reduce 2bc-DID alcohol drinking. Shell Ox1R blockage did not alter concurrent water intake in 2bc-DID **(E)** or 2bc-IA **(F)**; water intake indicated by cross-hatching. **(G)** Shell GABA-mediated inhibition did not alter concurrent water intake in 2bc-DID. M/B, muscimol/baclofen; SB, SB-334867. ^∗^*p <* 0.05, ^∗∗^*p* < 0.01.

Ox1R inhibition within the Shell, using SB-334867 (SB, 3-μg/side) (Hollander et al., 2008; Espana et al., 2010; James et al., 2011; Plaza-Zabala et al., 2012; Qi et al., 2013; Brown et al., 2015; Lei et al., 2016b), significantly reduced 2bc-DID alcohol intake (Figure 1A; *t*_13_ = 2.256, *p =* 0.0420) as previously observed (Lei et al., 2016b). Shell Ox1R inhibition, with the same SB dose, also significantly decreased intake during 2bc-IA intake (Figure 1B; *t*_29_ = 3.004, *p =* 0.0054; measured during the first 2 h of intake). SB at a 1-μg/side dose in Shell did not reduce 2bc-DID intake (*n* = 11; veh: 2.00 ± 0.31 g/kg intake; 1-μg SB: 1.78 ± 0.14 g/kg intake; *t*_10_ = 0.699, *p =* 0.5008), suggesting a dose-dependent effect. Thus, Shell Ox1Rs critically contributed to driving binge alcohol intake under both 2bc-DID and 2bc-IA models. To further understand how the Shell contributes to alcohol intake, we examined whether inhibiting the Shell with muscimol/baclofen (M/B, 50 ng of each/side), which produces more global inhibition of activity (Chaudhri et al., 2010; Millan et al., 2010; Wilden et al., 2014), would reduce 2bc-DID intake. Like Ox1R inhibition, GABA-mediated Shell inhibition also significantly reduced 2bc-DID alcohol intake (Figure 1C; *t*_10_ = 2.710, *p =* 0.0219). Because administration of Ox1R blocker into Shell could result in drug diffusion to the adjacent nucleus accumbens core (Core) and act there to inhibit intake, we also infused SB directly into the Core, which did not reduce 2bc-DID alcohol consumption (Figure 1D; *t*_9_ = 1.427, *p =* 0.1874). These results suggest that NAc Ox1Rs contributed to binge intake in a subregion-specific manner, and that Shell inhibition significantly reduced multiple forms of excessive alcohol consumption.

Reduced alcohol drinking after Shell inhibition could indicate more general motor and motivational effects. However, we previously showed that Shell Ox1R inhibition does not reduce saccharin intake (Lei et al., 2016b), and inhibition here of Shell Ox1Rs or general activity had no effect on concurrent water intake (Figure 1E–G; 2bc-DID SB: *p =* 0.6698; 2bc-IA SB: *p =* 0.2920; 2bc-DID M/B: *p =* 0.1563), similar to previous studies (see section “Discussion”). In addition, we observed no changes in preference (Table 2), perhaps due to the low level of concurrent water drinking across the 2-h session (Dhaher et al., 2009; Seif et al., 2015; Hartog et al., 2016; Lei et al., 2016b), although concurrent water intake was greater during 2bc-IA versus 2bc-DID sessions (*p =* 0.0032 Mann–Whitney) and 2bc-IA preference change was nearly significant (not shown). Thus, Shell Ox1Rs and activity promoted excessive alcohol drinking, rather than regulating intake more generally.

**Table 2:** Alcohol preference ratio, expressed as percentage from (volume alcohol)/(volume alcohol + volume water), at baseline and after inhibition of alcohol intake.

	Vehicle	Drug	Wilcoxon *p* =
2bc-DID Shell SB	92.9 ± 2.8	96.4 ± 2.6	0.4688
2bc-IA Shell SB	78.4 ± 4.5	68.8 ± 5.5	0.0559
2bc-DID Shell M/B	95.0 ± 2.9	85.3 ± 5.9	0.3125
Baclofen 5 mg/kg	94.6 ± 2.1	90.8 ± 2.9	0.2637
Baclofen 1 mg/kg	92.4 ± 2.0	93.3 ± 1.7	0.8169


*Lack of change likely reflects the lower water intake levels during the 2 h session.*

### Shell Inhibition by Ox1R Blockers or GABA Agonists Predominantly Decreases Alcohol Intake in Higher-Drinking Individuals

Since we had similar effects of Ox1R block in different alcohol drinking models, and congruent effects of more global Shell inhibition, we were in a unique position to aggregate these findings to examine whether the Shell is a critical region that promotes excessive intake in higher-drinking individuals. Thus, we generated a large, combined data set from data in Figure 1A–C and previous Shell Ox1R 2bc-DID results (Lei et al., 2016b). Importantly, we examined the relationship between basal intake levels (alcohol drinking on vehicle test days) and the consumption difference between drug and vehicle sessions. In this way, we could determine whether Shell inhibition had a greater impact on alcohol consumption in higher drinkers relative to more moderate drinkers. In addition, since mouse drinking exhibits variability, Shell inhibition experiments were compared with studies combined from ineffective drug treatments (which we call the control, no-change group, described in detail in Figure 4). This no-change group allows better understanding of the basal-intake/drug-effect relationship, including possible mathematical effects (in particular, where higher basal intake might be more likely to show a decrease, and vice versa).

**FIGURE 2:**
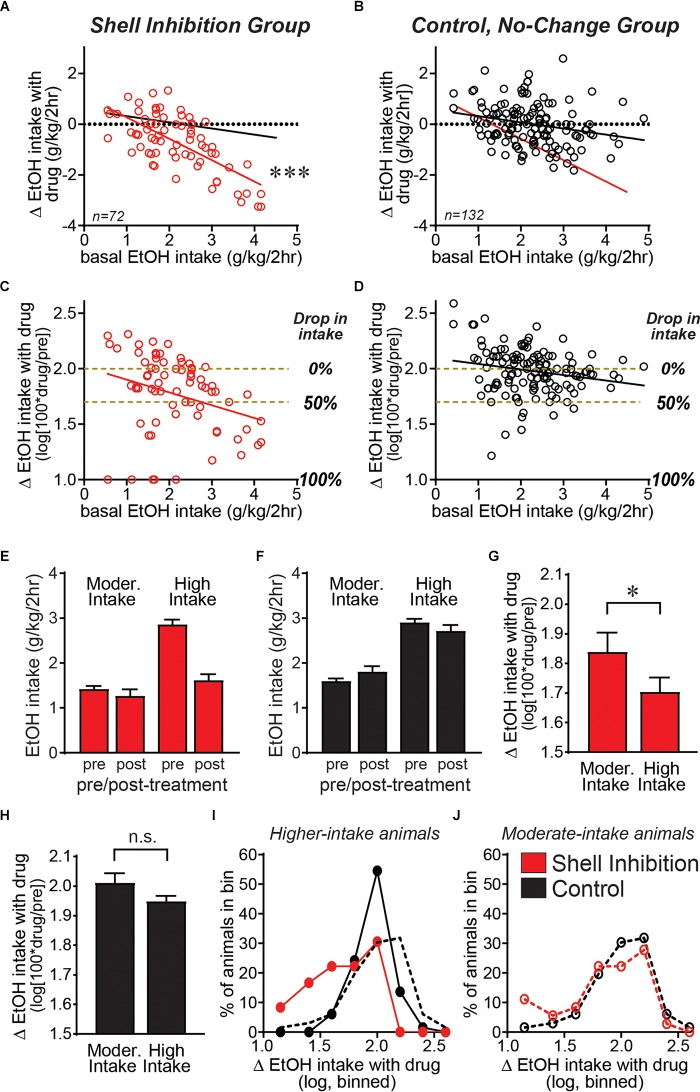
Shell Ox1Rs and activity were important for driving alcohol intake predominantly in higher-drinking individuals. Data combined from Shell inhibition groups **(A)** and separately combined from control (no-change) groups (where drug treatment had no impact on alcohol drinking) **(B)** examining how treatment-related drinking change relates to basal alcohol intake levels across individuals (basal consumption determined from vehicle-injected test sessions). The slope of the Shell Inhibition group (red line in **A,B**) was significantly greater than the slope in Control group (black line in **A,B**). **(C,D)** We next calculated the drug-related drinking change relative to basal intake levels in each animal: log[100^∗^(intake during drug treatment)/(intake during vehicle)]. Subjects with higher basal drinking showed a significantly greater impact of Shell inhibition, relative to individuals with more moderate basal consumption. Since the change in intake with treatment is on a log scale, yellow–brown dashed lines are included to indicate no change in drinking (0% drop in intake with treatment) compared with 50% drop in intake, and 100% drop in intake is also indicated. **(E–J)** A median split was used to divide individuals into moderate and higher basal drinkers (Moorman and Aston-Jones, 2009; Alcaraz-Iborra et al., 2017; Moorman et al., 2017). **(E,F)** Alcohol intake pre- and post-treatment in moderate versus higher basal drinkers, where Shell inhibition strongly reduced alcohol intake levels only in higher drinkers. **(G)** Shell inhibition produced a significantly greater decrease in alcohol drinking in higher-drinkers relative to moderate-drinkers. **(H)** In the control group, there was no difference in treatment-related change between higher and moderate basal drinkers. **(I,J)** Histograms of the number of mice showing different levels of change in drinking with treatment, binned as described in Section “Materials and Methods.” **(I)** In higher-drinkers, control mice (black) showed a normal distribution with a strong peak at log value of 2, indicating 100% of basal intake (no change). In contrast, higher-drinking mice with Shell inhibition (red) showed a clear shift to the left, indicating inhibition of alcohol consumption. **(J)** In moderate-drinkers, there was little difference in the distribution between Shell inhibition mice (red) and controls (black). ^∗^*p* < 0.05, ^∗∗∗^*p <* 0.001.

We first examined basal alcohol intake relative to the actual intake difference between drug and vehicle sessions; negative values indicate reduced consumption during drug relative to vehicle sessions. Figure 2A demonstrates that Shell Ox1R or GABAergic inhibition reduced alcohol drinking in individuals with higher basal drinking, with no overall effect in more moderate drinkers. Thus, basal drinking was significantly and negatively correlated with the change in drinking by Shell inhibition (*F*_1,70_ = 60.44, *p <* 0.0001). In the no-change group, basal drinking was also correlated with effect of drug treatment (Figure 2B; *F*_1,130_ = 7.077, *p =* 0.0088), an indicator that higher basal drinking is more likely to drop and lower intake likely to rise, separate from drug treatment (a mathematical artifact likely reflecting regression to the mean). Importantly, however, the slope of the Shell inhibition group (slope = -0.8445, *R*^2^ = 0.4633) was significantly larger than the slope of the no-change group (slope = -0.2428, *R*^2^ = 0.0516) (ANCOVA: Group: *F*_1,200_ = 37.044, *p <* 0.001; Basal intake: *F*_1,200_ = 44.656, *p <* 0.001; Group × Basal Interaction: *F*_1,200_ = 17.237, *p <* 0.001). These results suggest that Shell inhibition caused a greater disruption of alcohol intake in higher drinkers, indicating a more important role for Shell Ox1Rs in promoting excessive intake in higher-drinking individuals.

Since lower basal drinking limits the actual change in drinking levels, we next normalized the treatment-related intake change (or lack thereof) in relation to the basal consumption levels in each animal. In particular, we determined the percent change in drinking with treatment, and took the logarithm of this, determined as log[100^∗^(intake during drug treatment)/(intake during vehicle)] (see section “Materials and Methods”). Using this analysis, the Shell inhibition group still showed an overall similar distribution as in Figure 2A, with a bigger decrease in alcohol consumption with Shell inhibition in higher-drinking individuals (Figure 2C; *F*_1,70_ = 6.742, *p =* 0.0115, slope = -0.1155, *R*^2^ = 0.08786). In contrast, control animals showed much less difference between animals with moderate and higher intake (Figure 2D; *F*_1,130_ = 5.476, *p =* 0.0208, slope = -0.0499, *R*^2^ = 0.04042), although the slope was still significant, likely for mathematical reasons described above. We note that Shell inhibition did reduce drinking in some moderate-drinking subjects, and thus we performed several analyses to better understand Shell inhibition’s impact in subjects with different basal consumption. First, we performed a median split to divide animals into moderate versus higher drinkers (Moorman and Aston-Jones, 2009; Alcaraz-Iborra et al., 2017; Moorman et al., 2017). Figure 2E,F shows alcohol intake pre- and post-treatment in moderate versus higher drinkers, and demonstrate that Shell inhibition strongly reduced alcohol intake levels only in higher drinkers (Figure 2E), while treatment had no effect in the control group (Figure 2F). In addition, Shell inhibition caused a significantly greater decrease in alcohol intake in higher drinkers relative to moderate drinkers (Figure 2G; *p =* 0.0170 Mann–Whitney). In contrast, there was no difference in treatment effect between moderate and higher drinkers in the control (no-change) group (Figure 2H; *p =* 0.2102 Mann–Whitney). Thus, these results suggest that Shell Ox1Rs and activity played a stronger role in driving alcohol consumption in higher-drinking individuals.

We also generated histograms to examine the distribution of treatment effect across moderate and higher basal drinkers. In mice with higher basal intake (Figure 2I), control animals showed a normal distribution centered on log value of 2, indicating no change with treatment (100% of baseline). In contrast, higher-drinking Shell inhibition animals showed a clear shift to the left, indicating a significant decrease in intake compared with controls (*p <* 0.0001 Mann–Whitney). Mice with moderate basal intake (Figure 2J) had more subjects with increased intake, likely reflecting that drinking levels can increase more when starting from lower basal levels. In addition, there was no difference in the distribution of Shell inhibition and control moderate drinkers (*p =* 0.4925 Mann–Whitney). Together, these results confirm that Shell Ox1Rs and activity were essential for driving the excessive alcohol binging in higher drinkers, with limited contribution across moderate drinkers.

### 2bc-DID Alcohol Intake in Moderate Drinkers Can Be Reduced by GABA-B Receptor Activation

**FIGURE 3:**
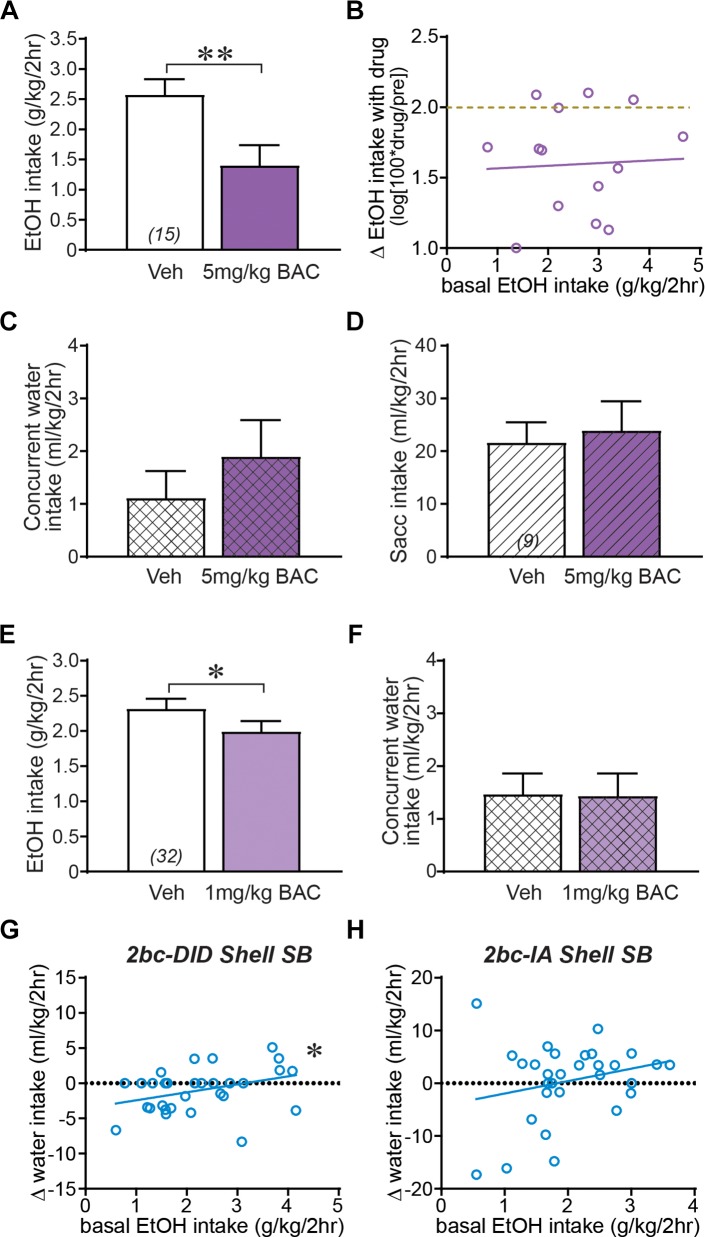
Shell regulation of alcohol drinking only in higher-drinking individuals was not due to a floor effect or other confounds. **(A)** 5 mg/kg baclofen (i.p.) significantly reduced 2bc-DID intake. **(B)** Baclofen reduction in alcohol drinking occurred in both moderate and higher basal drinkers. Dashed yellow–brown line indicates no effect of treatment on drinking level (0% drop in drinking). Baclofen inhibition of alcohol intake was not due to more general motivational or motor changes, since concurrent water intake **(C)** and saccharin intake tested in separate mice (**D,** diagonal lines) were not reduce by 5 mg/kg baclofen. 1 mg/kg baclofen slightly but significantly reduced alcohol intake **(E)** without altering concurrent water consumption **(F)**. **(G,H)** While concurrent water intake did not change during Shell Ox1R inhibition, higher basal alcohol intake was not accompanied by a larger decrease in water consumption for 2bc-DID intake (**F**, data from Figure 1E and Lei et al., 2016b) or 2bc-IA intake **(G)**. In fact, for 2bc-DID, higher basal intake was slightly but significantly correlated with greater water drinking. Together, these are further evidence that the selective effect of Shell inhibition in higher basal drinkers is not inexorably due to mathematical or other differences in higher or moderate intake individuals. BAC, baclofen. ^∗^*p <* 0.05, ^∗∗^*p* < 0.01.

The overall lack of effect of Shell inhibition in moderate drinkers could reflect a floor effect, where drinking could not be reduced further. This possibility was unlikely for several reasons. We examined the impact of the GABAB receptor agonist baclofen, which reduces alcohol intake in humans and animals (Mirijello et al., 2015; Bell et al., 2017). Baclofen (5 mg/kg, i.p.) significantly decreased 2bc-DID drinking (Figure 3A; *t*_14_ = 3.816, *p =* 0.0019), and, importantly, this was observed in both moderate and higher drinkers (Figure 3B), since the slope of basal intake versus treatment effect was nearly zero (slope = 0.0186, *F*_1,13_ = 0.0311, *p =* 0.8628). The baclofen reduction in intake was specific to alcohol, since 5 mg/kg baclofen did not reduce concurrent water intake during alcohol sessions (Figure 3C; *p =* 0.3594), and did not reduce saccharin consumption in separate mice (Figure 3D; *t*_8_ = 0.392, *p =* 0.7051). 1 mg/kg baclofen also slightly but significantly reduced alcohol drinking (Figure 3E; *t*_31_ = 2.257, *p =* 0.0312), without an effect on concurrent water intake (Figure 3F; *p* = 0.8987). Thus, baclofen produced a specific reduction in alcohol drinking that was similar across moderate and higher drinkers. In addition, to further rule out simple mathematical effects, we examined concurrent water intake, which was not reduced overall by Shell Ox1R inhibition (Figure 1D,E). If anything, Shell SB had the opposite on water intake relative to basal alcohol drinking levels, with a bigger drop in water intake in moderate basal drinkers; this was significant for 2bc-DID (Figure 3G; *F*_1,29_ = 4.456, *p =* 0.0435) although not 2bc-IA (Figure 3H; *F*_1,28_ = 1.822, *p =* 0.1879). Taken together, these results suggest that the minimal impact of Shell inhibition across moderate drinkers was highly unlikely to be due to a floor effect or other artifact (such as higher basal intake tending to go down, and lower to go up). Also, the median alcohol drinking level in the moderate drinkers was ∼1.55–1.65 mg/kg, and we previously showed that ∼1.6 g/kg/2 h leads to ∼80 mg% BACs (Lei et al., 2016a), suggesting that even moderate drinkers on average consumed binge alcohol levels. Thus, these findings together strongly support our hypothesis that Shell Ox1Rs and activity were specifically critical for driving the excessive binging in higher-drinking individuals.

### Generating the Combined Control Group, Where Treatments Did Not Alter Alcohol Drinking

**FIGURE 4:**
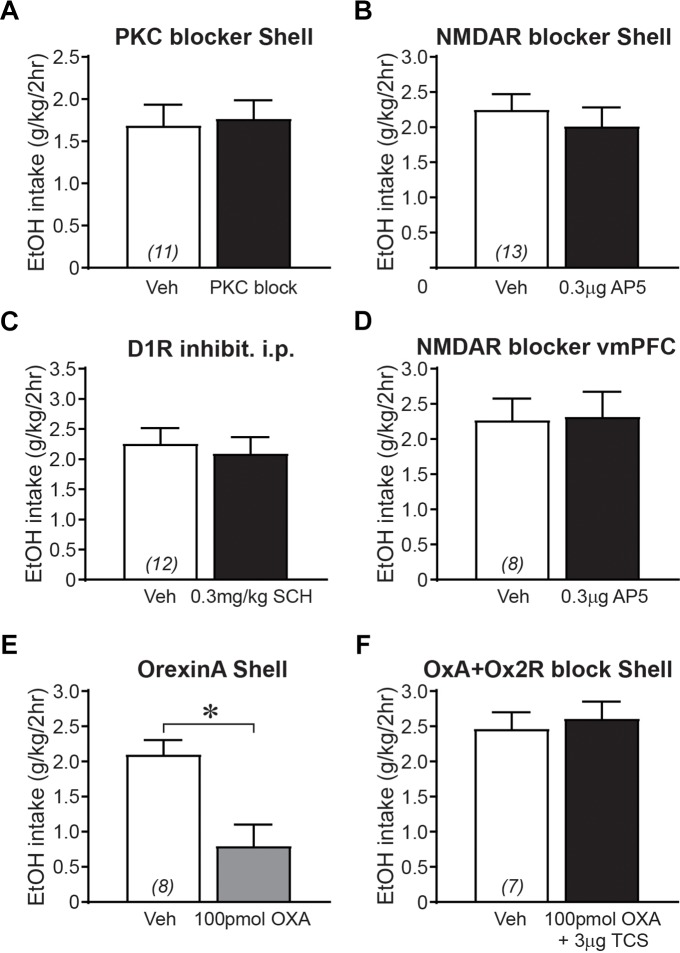
Control groups where 2bc-DID alcohol drinking was not altered by drug treatment. **(A)** Shell PKC inhibition did not alter 2bc-DID intake. **(B)** Shell NMDAR inhibition did not alter alcohol intake (combined 2bc-DID and 2bc-IA). **(C)** Systemic inhibition of D1Rs did not reduce 2bc-DID alcohol intake. **(D)** Inhibition of NMDARs in vmPFC did not reduce 2bc-DID alcohol consumption. OrexinA infusion in the Shell reduced alcohol intake **(E)** (combined 2bc-DID and 2bc-IA), which was prevented by co-infusion of the Ox2R blocker TCS **(F)**. 10 pmol/side orexinA also tended to decrease drinking (vehicle: 2.11 ± 0.30 g/kg; orexinA: 1.48 ± 0.12 g/kg; *p =* 0.0577, *n* = 4). ^∗^*p* < 0.05.

In addition to examining regulation of binge alcohol intake by Shell Ox1R- and muscimol-baclofen-sensitive activity, we also examined the importance of other signaling pathways which turned out to not alter intake; these became part of an aggregated control (no-change) group (as shown, e.g., in Figure 2B). First, evidence suggests that nicotine seeking is regulated by NAc Ox1Rs, PKC, and NMDARs (Plaza-Zabala et al., 2013). However, alcohol drinking was not impacted by inhibiting Shell PKC (Figure 4A; 0.03–0.4 μg/side chelerythrine; *t*_10_ = 0.325, *p =* 0.7521) or NMDARs (Figure 4B; 0.3 μg/side AP5; *t*_12_ = 1.036, *p =* 0.3205). Second, dopamine receptors are necessary for some alcohol-related behaviors (Hodge et al., 1997; Chaudhri et al., 2009; Pina and Cunningham, 2014; Hauser et al., 2015) but not others (Dickinson et al., 2003; Doherty and Gonzales, 2015), and systemic administration of the D1R blocker SCH23390 (0.3 mg/kg; Pina and Cunningham, 2014) did not alter intake (Figure 4C; *t*_11_ = 0.724, *p =* 0.4842). Third, since Shell Ox1R blockers decrease intake, we tested whether orexinA peptide (100 pmol/side) might increase drinking, but found that Shell orexinA actually decreased intake (Figure 4E; *t*_7_ = 4.188, *p =* 0.0041). In this regard, co-infusion of the Ox2r blocker TCS-OX2-29 (TCS, 3 ug/side, see Lei et al., 2016b) prevented the orexinA reduction in intake (Figure 4F; *t*_6_ = 0.793, *p =* 0.4580). Shell Ox2rs do not regulate alcohol drinking (Lei et al., 2016b), but these results are consistent with Shell orexinA enhancing locomotion through Ox2rs but not Ox1Rs (Thorpe and Kotz, 2005; Kotani et al., 2008) (and we speculate that increased locomotion could disrupt focus on alcohol drinking). Finally, we previously showed that vmPFC Ox1Rs promote binge alcohol drinking in addition to Shell Ox1Rs (Lei et al., 2016b). However, NMDAR block with AP5 within vmPFC did not reduce alcohol drinking (Figure 4D; *t*_7_ = 0.143, *p =* 0.8901). Together, these findings suggest that Shell PKC and NMDARs, vmPFC NMDARs, and D1Rs (tested systemically), were not critical for driving alcohol consumption. Thus, in order to construct the large no-change group to compare with our aggregated Shell inhibition group (Figure 2), results in Figure 4A–D,F were combined with data from no-change groups whose average values were previously reported (from Lei et al., 2016b: intra-Shell TCS, intra-Insula SB; from Lei et al., 2016a: systemic vehicle treatment during alcohol-only or quinine-alcohol drinking, 0.3 mg/kg SB for alcohol-only drinking, and systemic TCS for alcohol-only drinking).

### First Day Intake Predicts Consumption in Subsequent Weeks

**FIGURE 5:**
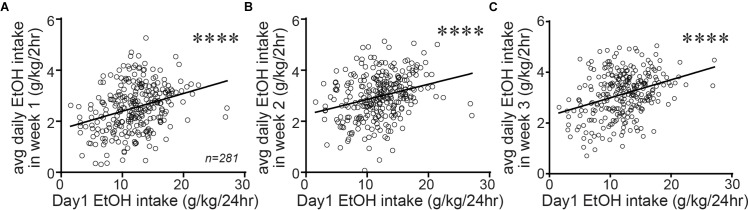
Alcohol drinking on the first day of intake predicted consumption 3 weeks later. Alcohol drinking level on the first day of intake (a 24 h session) significantly predicted the average 2 h intake during week 1 **(A)**, week 2 **(B)**, and week 3 **(C)** of 2bc-DID. This suggests that the basal alcohol consumption level may reflect a more stable trait within each individual, although the variability in mice indicates that some caution is warranted in this interpretation. ^∗∗∗∗^*p <* 0.0001.

Since Shell Ox1R and activity were critical for driving alcohol drinking in animals with higher basal intake, it would be particularly interesting to determine whether higher basal intake reflected a trait within particular individuals. Again, taking advantage of the large data set that we possess (*n* = 281), we examined whether alcohol drinking on the first day of access (the initial 24 h intake session in 2bc-DID drinkers) predicted 2 h intake levels averaged within each week of 2bc-DID intake. In fact, the initial-day drinking significantly predicted subsequent 2bc-DID intake levels on week 1 (Figure 5A; *F*_1,279_ = 30.65; *p* < 0.0001; *R*^2^ = 0.099), week 2 (Figure 5B; *F*_1,279_ = 24.52; *p* < 0.0001; *R*^2^ = 0.081), and week 3 (Figure 5C; *F*_1,279_ = 35.11; *p* < 0.0001; *R*^2^ = 0.112). Also, alcohol intake slightly but significantly increased across the first 3 weeks of 2bc-DID consumption (week 1: 2.535 ± 0.054 g/kg/2 h; week 2: 2.976 ± 0.052 g/kg/2 h; week 1: 3.139 ± 0.052 g/kg/2 h; *F*_2,280_ = 57.78, *p* < 0.0001 one-way RM ANOVA; *p* < 0.01 difference between intake on any pair of weeks). Our results support the possibility that inter-individual variation in drinking levels is a trait, although there is some variability in the data that make it possible that some individuals increase or decrease intake across weeks of drinking. Thus, basal intake for all other studies was determined later in intake, nearer to the actual test sessions.

## Discussion

**FIGURE 6:**
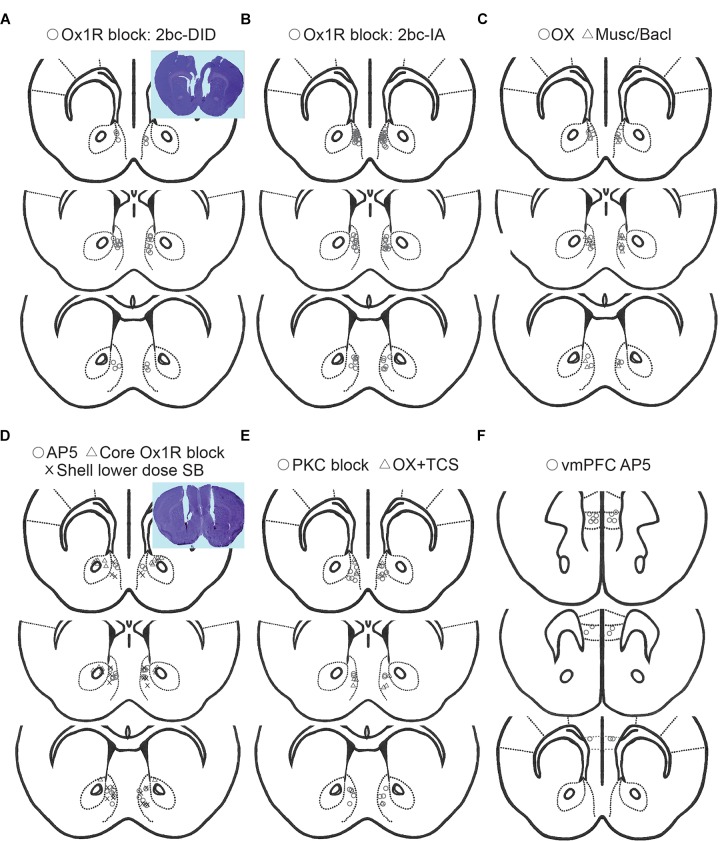
Histology of cannulae placements. Placements for 2bc-DID Shell SB (**A**, circles), 2bc-IA Shell SB (**B**, circles), Shell muscimol/baclofen (**C**, triangles), Shell orexin (**C**, circles), Core SB (**D**, triangles), Shell AP5 (**D**, circles), Shell 1 μg-side SB (**D**, X), Shell PKC block (**E**, circles), orexin+TCS (Ox2R blocker) (**E**, triangles), vmPFC AP5 (**F**, circles). All placements are for Shell, except for Core and vmPFC as specifically indicated.

Excessive, binge alcohol drinking is a significant obstacle to treating AUD. Importantly, heavy-drinking individuals account for much of the substantial costs and harms of AUD, making it critical to uncover the underlying mechanisms that drive this excessive consumption, since this may aid development of novel translational AUD interventions. Studies with systemic inhibitors suggest that Ox1Rs drive the higher alcohol intake in excessive-drinkers, but little has been known about the circuits where Ox1Rs promote this higher consumption. Here, we show that Shell (Figure 6) Ox1Rs promoted intake during both intermittent-access (2bc-IA) alcohol consumption as well as a two-bottle-choice Drinking-in-the-Dark (2bc-DID) model. Shell inhibition with muscimol/baclofen also decreased 2bc-DID intake. Using this large data set, we were able to show that the excessive intake in higher-drinkers was highly dependent on Shell Ox1Rs and activity, while alcohol consumption in moderate drinkers was largely unaffected by Shell inhibition. Ox1R promotion of alcohol drinking was site-specific, as inhibiting Core Ox1Rs did not significantly reduce intake. Also, alcohol drinking was not regulated by NMDAR or PKC inhibition in Shell or by systemic inhibition of dopamine-1 receptors. We combined results from these and other experimental groups exhibiting no change with treatment, and this control group was used to indicate that the selective importance of Shell Ox1Rs and activity within higher-drinking individuals was not simply a reflection of intrinsic variability of mouse drinking. Furthermore, systemic baclofen reduced alcohol drinking regardless of basal intake levels, without altering concurrent water intake or saccharin consumption, indicating that the lack of effect of Shell inhibition in moderate-drinkers was not due to a floor effect on drinking suppression (inability to reduce intake). Finally, initial day drinking was significantly correlated with alcohol consumption weeks later, supporting the possibility that basal alcohol intake is a stable trait within each individual. Together, our studies strongly suggest that Shell Ox1Rs are critical mediators of the excessive binge intake in higher-drinking individuals, and that targeting Ox1Rs may substantially reduce AUD-related harms.

Ox1Rs are important for driving seeking and intake of motivating natural rewards as well as intoxicants such as cocaine, opioids, nicotine and alcohol, but play little role for consumption of less motivating substances (Borgland et al., 2009; Cason et al., 2010; Baimel et al., 2014; Mahler et al., 2014). For example, Ox1Rs mediate the increased alcohol consumption observed in alcohol-dependent mice (Lopez et al., 2016) as well as the high intake in genetically alcohol-preferring rats (Anderson et al., 2014). In addition, systemic inhibition of Ox1Rs has suggested that Ox1Rs play a much greater role in driving drinking in individuals with higher basal alcohol intake levels (Moorman and Aston-Jones, 2009; Alcaraz-Iborra et al., 2017; Moorman et al., 2017), but, prior to our studies, the region where Ox1Rs act to promote high drinking in excessive individuals had remained unknown. Thus, ours is the first demonstration that Ox1Rs in Shell played a critical role in driving alcohol consumption in heavy drinkers, suggesting that they may be a potent target for reducing harms associated with human heavy drinking. We also note that we found no effect of PKC or NMDAR inhibition in Shell, or NMDAR block in vmPFC, on excessive alcohol drinking. We initiated these studies because nicotine seeking may be regulated by NAc Ox1Rs, PKC, and NMDARs (Plaza-Zabala et al., 2013). However, other studies have not found a role for NAc NMDARs for driving alcohol drinking (Eisenhardt et al., 2015; see Hopf, 2017). Similarly, D1Rs can regulate some alcohol behaviors (see above), but not others (Dickinson et al., 2003; Doherty and Gonzales, 2015). These negative studies limit the possible receptor and signaling pathways that mediate or interact with Ox1Rs to drive alcohol binge intake, and provide important information to focus future studies on the molecular pathways through which Ox1Rs in Shell (and vmPFC) drive excessive alcohol intake.

OxRs can play a more general role in feeding and arousal (Mahler et al., 2014; Brown et al., 2015; Li et al., 2016), and the Shell, including OxRs, can regulate feeding (Thorpe and Kotz, 2005; Urstadt and Stanley, 2015; but see Baldo and Kelley, 2001). Thus, reduced alcohol drinking with Ox1R inhibition might be secondary to decreased motivation or motor activity. However, inhibition of Shell Ox1R or activity did not change concurrent water intake. In addition, systemic baclofen did not reduce concurrent water intake or saccharin consumption. These findings agree with other studies where altering Shell activity suppresses intake of alcohol but not sweet fluid (Stratford and Wirtshafter, 2011; Rewal et al., 2012; Kasten and Boehm, 2014; Lum et al., 2014). Similarly, Shell Ox1R inhibition does not impair chow intake or locomotor activity (Thorpe and Kotz, 2005; Kotani et al., 2008; Qi et al., 2013). We note that this is in contrast to systemic application of OxR blockers, which can reduce intake of sweetened solutions (Anderson et al., 2014; Olney et al., 2015; but see Lopez et al., 2016). Nonetheless, inhibiting Ox1Rs likely reflects an important intervention that could suppress the excessive intake in higher-drinkers.

Our studies strongly implicate Shell Ox1Rs and activity as essential drivers of the higher intake levels in excessive-drinking individuals. More generally, the Shell has been implicated in a number of different addiction-related and consummatory behaviors, including control of feeding (Baldo et al., 2013; Richard et al., 2013) and alcohol consumption (Kasten and Boehm, 2014; Lum et al., 2014; Wilden et al., 2014; Ramaker et al., 2015). The Shell also mediates reinstatement behaviors involving alcohol (Chaudhri et al., 2010; Marchant et al., 2015) and other drugs (Anderson et al., 2008). However, other studies have shown that Shell suppresses responding under some conditions, especially under learned extinction (Peters et al., 2009) including for alcohol (Millan et al., 2010). This may reflect the extinction condition, since Shell promotes opiate reinstatement behaviors (Bossert et al., 2015; Hearing et al., 2016), including where Shell OxRs mediate reinstatement of morphine place preference (Qi et al., 2013; Sadeghzadeh et al., 2016). Thus, there is considerable precedent for Shell promoting addiction-related behavior, although it can play other roles under some conditions, and these studies overall are in agreement with our demonstration that Shell Ox1Rs and activity are critical for driving excessive intake in higher-drinking individuals.

Across a large sample size, we find a significant correlation between alcohol drinking levels on the first day of intake and consumption several weeks later. Although there is some variability in the data, these results suggest that basal intake levels may be a more stable trait within each individual. Other studies are consistent with this possibility. For example, Wolstenholme et al. (2011) found individual differences in alcohol drinking that were consistent across the three weeks of drinking, which relate to genetic differences across subjects (see also Mulligan et al., 2011). These findings suggest that individual differences in intake reflect a more consistent trait within each subject, and it will be interesting in future studies to investigate the mechanistic nature of the differential Shell Ox1r contribution in higher-drinking versus moderate individuals.

Other studies of individual differences across mice have observed interesting behavioral patterns that relate to drive for drinking. Anxiety and negative affect can be important drivers of alcohol consumption (Koob and Volkow, 2010), and higher-drinking individual mice have higher anxiety and compulsion-like alcohol responding, with no difference in saccharin preference (Radwanska and Kaczmarek, 2012; Bahi, 2013). In addition, these alcohol intake differences across individuals are not related to variation in sweet and bitter taste reactivity (Wolstenholme et al., 2011; Radwanska and Kaczmarek, 2012; Bahi, 2013), and are thus more specific to alcohol-related behavior. High-binging mouse strains have also been extensively studied (e.g., Phillips et al., 2010; Vanderlinden et al., 2015), and high binging is associated with more impulsivity, similar to what is observed in human drinkers (Sanchez-Roige et al., 2014). Thus, there are individual differences across mice related more selectively to pathological alcohol behaviors, including higher intake and comorbid contributors, and our large data sets have provided findings that give Shell Ox1Rs a prominent role in driving the excessive alcohol intake levels in higher drinkers. Interestingly, only a subset of humans that drink alcohol go on to exhibit binge drinking and other pathological intake behaviors (Hopf and Lesscher, 2014; Augier et al., 2018), so NAc Ox1rs might represent an important risk factor for heavy drinking in vulnerable individual humans.

We chose the 3-μg/side dose of SB-334867 here since it has been used in many recent studies that observe selective behavioral effects. Importantly, we find that Shell SB has no impact on saccharin intake but does significantly reduce alcohol drinking (Lei et al., 2016b). In agreement, this SB dose in Shell does not impact locomotor activity or drug-prime reinstatement of morphine CPP, although it does decrease stress-induced reinstatement (Qi et al., 2013). Thus, while we did not examine locomotor or food intake here, findings from our group and others suggest that Shell SB changes in alcohol drinking observed here (and in Lei et al., 2016b) do not reflect reduced ability to seek or consume. In addition, a comparable SB dose in cortical areas reduces nicotine but not food intake (Hollander et al., 2008) and alcohol but not sucrose seeking (Brown et al., 2016), and this dose reduces alcohol seeking and cocaine intake but not locomotion in VTA (James et al., 2011; Brown et al., 2016). Furthermore, i.c.v. administration of a similar dose decreases alcohol but not saccharin or food intake (Carvajal et al., 2015). Thus, the behavioral selectively of our SB dose used has been widely examined. In addition, we found here that 1-ug/side SB in the Shell did not reduce DID alcohol intake, indicating a dose-dependent effect of Shell Ox1R inhibition on alcohol drinking.

Finally, we also note that the importance of Ox1Rs may be behavior specific, and thus different SB doses may be effective under different conditions (e.g., chow verse alcohol). In our work, we find that low doses of SB (given i.p.) strongly reduce compulsion-like alcohol drinking (Lei et al., 2016a), relative to the higher SB doses needed to decrease non-compulsion-like intake (Anderson et al., 2014; cf. Lei et al., 2016a). While many behaviors require higher systemic doses to inhibit behavior (Mahler et al., 2012, 2014; Anderson et al., 2014; Moorman et al., 2017), studies using lower SB doses find efficacy against alcohol intake in alcohol-preferring rats (Anderson et al., 2014; Moorman et al., 2017), nicotine intake (Hollander et al., 2008), and stress-induced reinstatement (Richards et al., 2008), which all likely reflect states of high motivation, similar to compulsion. Thus, different motivational contingencies might influence the impact level of orexin signaling on behavior.

## Conclusion

Heavy-drinking individuals are responsible for much of the substantial costs and harms of AUD, and our results strongly suggest that Shell Ox1Rs are critical drivers of the high alcohol consumption levels in excessive-drinking individuals. Using large, combined data sets, we compared the impact of Shell inhibition on alcohol intake with the consumption pattern seen in an aggregated control group, which allowed us to account for intrinsic variability of mouse drinking. Furthermore, systemic baclofen reduced intake in higher and moderate-drinkers, suggesting that the overall lack of effect of Shell inhibition in moderate drinkers did not reflect a floor effect. Finally, initial drinking strongly predicted alcohol consumption in subsequent weeks, suggesting that basal intake level was a more stable trait in each subject. Thus, our results support the possibility that targeting Ox1Rs in higher-drinking individuals could substantially decrease the overall negative impact of AUD.

## Author Contributions

KL, CK, JY, SW, LN, SG, VP, LA, and MF performed the experiments. KL, DD, and FH performed the analyses. KL and FH prepared the manuscript.

## Conflict of Interest Statement

The authors declare that the research was conducted in the absence of any commercial or financial relationships that could be construed as a potential conflict of interest.
